# Kinetics of IgG antibody to cytomegalovirus (CMV) after birth and seroprevalence of anti-CMV IgG in Chinese children

**DOI:** 10.1186/1743-422X-9-304

**Published:** 2012-12-10

**Authors:** Jie Chen, Lingqing Hu, Meiling Wu, Tianying Zhong, Yi-Hua Zhou, Yali Hu

**Affiliations:** 1 Department of Obstetrics and Gynecology, Nanjing Drum Tower Hospital, Nanjing Medical University, Jiangsu, China; 2 Department of Obstetrics and Gynecology, Wuxi Maternal and Child Health Hospital, Jiangsu, China; 3 Department of Laboratory Medicine, Nanjing Children’s Hospital, Nanjing Medical University, Jiangsu, China; 4 Departments of Experimental Medicine and Infectious Diseases, Nanjing Drum Tower Hospital, Nanjing University Medical School, Jiangsu, China; 5 Jiangsu Key Laboratory for Molecular Medicine, Nanjing University Medical School, Jiangsu, China

**Keywords:** Cytomegalovirus, Anti-CMV IgG, Kinetics, Primary infection, Children

## Abstract

**Background:**

Prevalence of cytomegalovirus (CMV) infection is 90–100% in developing countries; however, the kinetics of anti-CMV IgG in infants remains elusive.

**Methods:**

Sera from 112 mother-newborn pairs and longitudinal samples from 41 infants up to 2-year old were tested for anti-CMV IgG and IgM. Additionally, samples from 837 healthy children were included.

**Results:**

Of 112 mothers, 108 (96.4%) were anti-CMV IgG positive; their 108 newborns were also seropositive. In a 2-year follow-up among 40 infants of positive mothers, anti-CMV IgG level in 8 individuals decreased with time and became undetectable by age of 3.5–8 months, and that in 32 others decreased at 1- and 3.5-month old, and then increased. Based on the positive IgM, rising IgG levels, and low anti-CMV IgG avidity index, 76.7% of the primary infections were demonstrated to occur during 1–3.5 months of age. The overall seroprevalence of anti-CMV in 837 children was 82.4%, which was generally constant from 2 to 8 years old (χ^2^ = 3.150, *p* = 0.790).

**Conclusions:**

The maternally acquired anti-CMV IgG in infants disappears before 8-month old. Primary CMV infection in Chinese children mostly occurs during 1–3.5 months of age. Whether the relatively lower seroprevalence of anti-CMV in Chinese children found in this survey may reflect the positive rate in child-bearing age women in the future remains to be further studied.

## Background

Human cytomegalovirus (CMV) infection is ubiquitous throughout the world. It is well documented that the seroprevalence of CMV in young women of childbearing age is 40–80% and 90–100% in developed and developing countries respectively [1]. CMV is the most common cause of viral intrauterine infection, with an incidence of 0.03–2.0% in all live births [1,2]. Congenital CMV infection can cause birth defects such as mental retardation, and increase the risk of morbidity and mortality in newborns [3-5]. Studies demonstrated that preconception maternal immunity to CMV provides substantial protection against vertical transmission and decrease the incidence of diseases and permanent physical sequelae in infants [6,7]. Although various mechanisms may account for these findings, an important explanation is the transplacental immunity mediated by maternal IgG antibodies in the fetus [8]. To date, the immunologic events after transplacental transfer of maternal anti-CMV IgG in infants are poorly characterized. In addition, although as high as 90–100% women in developing countries are anti-CMV IgG positive, the age of primary CMV infection in general population remains elusive.

In the present study, we compared the anti-CMV IgG concentrations between maternal and cord sera to characterize transplacental transfer of maternal anti-CMV IgG. Furthermore, we studied the evolution of anti-CMV IgG in the longitudinal serum samples within two years after birth and determined the positive rate of anti-CMV IgG among children by different ages.

## Results

### Transplacental transfer of maternal anti-CMV IgG

**Figure 1:**
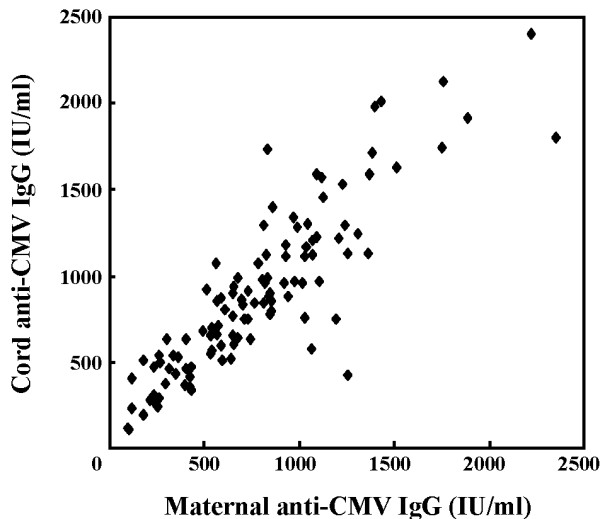
**Correlation of transplacentally transferred anti-CMV IgG in infants with the maternal antibody.** Anti-CMV IgG level in the cord blood was correlated with that in mothers (linear regression analysis, *y* = 1.04*x* + 105.09, r = 0.87, *p* < 0.001, n = 108).

Of the 112 mothers, 108 (96.4%) were anti-CMV IgG positive. To investigate transplacental transfer of maternal anti-CMV IgG, we quantified the IgG in 108 mother-newborn pairs. Each mother transferred anti-CMV IgG to her newborn, because the IgG was positive in each cord serum. The geometric mean concentration (GMC) of anti-CMV IgG in the cord blood (770.3 IU/ml, range 106.1–2407.3) was higher than that in the mother (654.2 IU/ml, 96.1–2351.7). In details, 75% of the cord sera had higher concentrations than the corresponding maternal sera (Table 1). Anti-CMV IgG level in the cord blood was correlated with that in mothers (Figure 1). The pregnant woman’s age, child’s age, and body weight were not correlated with the transplacental transfer efficiency (data not shown).

**Table 1:** Comparison of anti-CMV IgG between newborns and mothers

**Ratio of cord/maternal anti-CMV**	**No. (%)**	**Anti-CMV IgG (IU/ml)**				***p***
		**Mothers**		**Newborns**		
		**GMC**^**a**^	**Range**	**GMC**	**Range**	
≥1.5	15 (13.9)	349.1	113.2–855.8	683.2	230.4–1728.9	<0.001
1.01–1.49	66 (61.1)	686.0	96.1–2219.6	826.8	106.1–2407.3	<0.001
1	2 (1.9)	744.0	650.1–851.3	744.0	650.1–851.3	-
0.30–0.99	25 (23.1)	832.7	252.5–2351.7	688.9	240.3–1796.2	0.001
Total	108 (100)	654.2	96.1–2351.7	770.3	106.1–2407.3	<0.001

^a^GMC is the abbreviation of geometric mean concentration.

### Dynamic changes of anti-CMV IgG and IgM after birth

Forty-one children with longitudinal serum specimens were retrospectively measured for anti-CMV IgG and IgM. In one child of a mother with negative anti-CMV IgG, anti-CMV IgG and IgM were persistently negative in the cord blood and during the 24-month follow-up; we excluded this child in the further analysis. Thus, 40 mother-child pairs were included in the analysis of the dynamic changes of maternal anti-CMV IgG. The GMC of anti-CMV IgG in the mothers and newborns was 759.8 IU/ml (232.1–2351.7) and 976.5 IU/ml (227.3–1935.0), respectively.

Of these children, the IgG concentration in 8 individuals decreased with time and became undetectable as early as 3.5–8 months after birth (Table 2), demonstrating that the maternally acquired anti-CMV IgG in infants disappears before 8-month old. In 32 others, the IgG levels decreased before 3.5-month old, but significantly increased at 8-month old, and slightly decreased at 2 years of age (Table 2). The GMC of anti-CMV IgG in the 8 and 32 corresponding mothers was 672.4 IU/ml (264.2–1753.6) and 791.4 IU/ml (232.1–2351.7), respectively (*t =* 0.354, *p =* 0.726). The profile of the IgG level after birth was further analyzed between the two groups (Table 2). At both 0- and 1-month-old, the IgG levels in two groups were not statistically different and decreased in a similar extent. However, at 3.5-month-old, the reduction of the IgG levels was less obvious in the 32 children. Thereafter, the IgG levels increased significantly in the 32 children, but became persistently negative in the 8 children.

**Table 2:** Kinetics of anti-CMV IgG level in infected and uninfected children after birth

**Age (month)**	**Anti-CMV IgG (IU/ml)**					**Statistics**
		**Infected children** **(n = 32)**		**Uninfected children** **(n = 8)**			
	**GMC**^**a**^	**Range**	**GMC**	**Range**	***t***	***p***	
0	856.2	227.3–1935.0	843.5	321.3–1602.9	0.089	0.930
1	575.0	249.2–1241.3	454.7	112.2–1006.0	0.528	0.603
3.5	395.4	129.4–1162.3	75.8	7.0–507.9	2.641	0.016
8	984.5	299.6–1986.0	Negative	–	–	–
24	637.3	240.7–1902.8	Negative	–	–	–

^a^GMC is the abbreviation of geometric mean concentration.

None of the 112 cord blood showed positive for anti-CMV IgM. Anti-CMV IgM was also tested in 30 infected individuals with sufficient samples (Table 3). The IgM was positive in 27 children and was persistently negative in longitudinal sera of 3 other individuals. Together with the anti-CMV IgG kinetics (Table 2), the results strongly suggested that CMV infection was not congenitally, but postnatally, acquired.

**Table 3:** Age distribution of anti-CMV IgM in 30 infected children

**Age (month)**	**Positive no.**	**Positive rate (%)**
1	4	13.3
3.5	21	70
8	2	6.7
24	0	0
Total	27	90

### Seroprevalence of anti-CMV among children at different ages

A total of 837 sera from 837 children (513 males) aged 0–8 years were tested for anti-CMV IgG. The IgG was positive in 690 children and negative in 147 others. The overall positive rate of anti-CMV IgG was 82.4% (83.2% in boys and 81.2% in girls, χ^2^ = 0.584, *p =* 0.445). Generally, the positive rate slightly varied among the different age groups. As shown in Table 4, anti-CMV IgG positive rate was highest in infants <6-month old, decreased significantly to 73.4% in the 6–12-month age, and increased to 83.0% in the 1–2-year age (χ^2^ = 13.419, *p =* 0.001). Then, the seroprevalence showed no significant fluctuation until 8-year old (χ^2^ = 1.212, *p* = 0.796).

**Table 4:** Age distribution of anti-CMV IgG seropositive children

**Age group (year)**	**Total no.**	**Positive no. (%)**
<0.5	92	86 (93.5)
0.5–1	94	69 (73.4)
2	94	78 (83.0)
3	94	77 (81.9)
4	93	75 (80.6)
5	91	75 (82.4)
6	90	77 (85.6)
7	94	77 (81.9)
8	95	76 (80.0)
Total	837	690 (82.4)

Since anti-CMV IgG may be negative but IgM is positive in the very early infection period, we tested anti-CMV IgM in the 147 IgG negative children to clarify whether some children were in this period; none of the 147 children was the IgM positive. To define whether the anti-CMV IgG positive children were actively or latently infected, we tested anti-CMV IgM in 280 samples with the IgG concentration >500 IU/ml, and found that 23 (8.2%) were anti-CMV IgM positive; they were all bellow 6-year old and 12 (52.2%) were under 1-year old, with the youngest only 1.5 months of age.

### Primary CMV infection in childhood

Based on the profiles of anti-CMV IgG in the infants and children, primary CMV infection appears to occur mainly in the first year of life. Thus, we further tested CMV IgM in all infants under 1-year old; the overall positive rate was 15.6% (29/186), higher than that in the other age groups. Moreover, the CMV IgG avidity assay was performed in the IgM positive individuals. On the whole, CMV IgG avidity assay results indicated low AI (median 23.6%, range 10.3–52.1%) in infants aged 3–12 months, whereas intermediate or high AI (42.9%, 36.5–58.3%) in the 1–3-month and 1–6-year age (62.8%, 14.8–87.6%).

In the retrospective study, as shown in Table 3, the positive rate of anti-CMV IgM was highest (70%) during 1–3.5 months of age and the IgM was persistently negative in longitudinal sera of three individuals. In 2 of the IgM negative infants, the concentration of anti-CMV IgG decreased from 524.3 and 647.5 to 249.2 and 278.2 IU/ml at 1-month-old, respectively, and then increased to 348.0 and 381.3 IU/ml at 3.5 months of age, respectively. Therefore, the primary CMV infection occurred before 3.5-month old in these two infants although they were IgM negative, which may be related to the fact that the IgM assay may lack sensitivity. In another one, the IgG was negative at 8-month-old and seroconversion was observed at 2-year old, suggesting a primary CMV infection occurred during 8–24 months old. Together with above two infants with increased anti-CMV IgG at 3.5-month old, totally 76.7% (23/30) underwent primary CMV infection during 1–3.5 months of age. CMV IgG avidity assay also showed that all primary infected infants had CMV IgG AI <30%.

## Discussion

In the present study, we demonstrate that anti-CMV IgG in pregnant women can efficiently transfer to their fetuses and the maternal anti-CMV IgG in the majority of newborns is higher than that in their mothers. Moreover, we validate that primary CMV infection in children in China mainly occurred during 1–3.5 months of age.

Transplacental transfer of maternal IgG to the fetal bloodstream is mediated by neonatal Fc receptor in syncytiotrophoblasts of the placenta and contributes to the passive immunity of newborns to pathogens. Maternal IgG antibodies in full-term newborns are usually higher than those in their mothers [9]. In agreement with previous studies [10,11], we found that anti-CMV IgG in mothers transferred to the fetuses and anti-CMV IgG in most cord sera exceeded the maternal levels. Furthermore, in the present study, we found that the seroprevalence of anti-CMV IgG was much higher in young infants (<6 months old) as compared to older children (Table 4), which is likely to be related to the persistence of maternal antibodies in this group. Similarly, the intermediate or high avidity antibodies detected in infants <3 months of age may be explained by the transplacental transfer of maternal antibodies.

Transplacentally acquired maternal antibodies may protect infants against diseases in the early period of life. In the present study, despite maternal anti-CMV IgG in infants, primary CMV infection occurred early in childhood, indicating that maternal anti-CMV IgG can not fully protect against CMV infection. On the other hand, in spite of being infected, the children showed no symptoms related with CMV infection. Thus, maternal anti-CMV IgG in infants may provide substantial protection against symptomatic diseases or sequelae. This is similar to the prophylactic purpose of hepatitis A vaccination among children [12].

In developing countries, the incidence of primary CMV infection has been reported to peak predominantly in the first years of life [13,14]. However, in developed countries, it occurs not only nearly at the end of childhood [15], but also in women of child-bearing age [16]. In our study, based on the kinetics of anti-CMV IgG (Table 2), the seroconversion of anti-CMV IgM (Table 3) and the results of CMV IgG avidity assay in children with longitudinal sera, we concluded that primary CMV infection in children mainly occurred before 3.5 months of age, which is earlier than the infection age identified before in developing countries [13,17]. For the children in the second group, only cross-sectional serum samples were available. It is possible that some CMV-infected children did not develop detectable specific antibodies. Therefore, some antibody negative infants may actually have been infected. However, we consider that the proportion of CMV IgG negative infected children should be very small since the CMV IgG positive rates in children at same age were similar in the retrospective and cross-sectional groups (Table 4).

In the present study, the seroprevalence in the children aged 0–8 years was around 80% and generally constant from 2 to 8 years old (Table 4), which indicates that there may be little CMV infection taking place between 2 and 8 years of age in this population. Also, we found that 96.4% of the pregnant women were anti-CMV IgG positive, which is in agreement with the rate of anti-CMV IgG (93–98%) in child-bearing age women in China [17,18]. The difference of the two rates may be due to the variation in populations with different ages since CMV infection through sexual contact is likely to increase seroprevalence by the time of adulthood. Alternatively, the prevalence in the children may reflect the positive rate in child-bearing age women in the future, since social economic situation and hygienic status in China have been greatly improved. However, with no previously reported prevalence of anti-CMV IgG in Chinese children, it is difficult to determine which is more reasonable to explain the reduced prevalence in the children. Therefore, further research is needed to clarify this issue.

Our findings that most CMV infection in children occurred before 3.5-month old also suggest that the CMV infection source in children is mainly from mothers or other family members since such young infants rarely contact others in China. The results that one child of the mother with negative anti-CMV IgG was persistently negative for anti-CMV IgG also support the assumption. Indeed, CMV in body fluids, such as breast milk may be associated with the risk and severity of postnatal CMV infection [19-21].

There are several limitations in the present study. First, we defined the CMV infection in infants based on the dynamic changes of anti-CMV IgG, which was quantitatively tested only using the Dia.Pro test system. Since the dynamic ranges of different IgG ELISAs are highly different, it would have been helpful to compare the results of the Dia.Pro test system with other frequently used ELISA system. However, in our study, the sera from the mother-newborn pairs and longitudinal follow-up were tested in parallel in each assay. Additionally, the inter- and intra- assay variations were well within acceptable limits. Second, the gold standard for the diagnosis of CMV infection in early life is to detect the virus in urine or saliva by viral culture and/or PCR. As our study was retrospective, we did not have children’s urine or saliva samples and only had children’s serum samples; PCR has relatively lower sensitivity in detecting CMV DNA in serum [22]. Thus, we did not perform viral culture and PCR to diagnose CMV infection. Third, 13.3% of the infants at the age of 1 month (Table 3) were seroconverted to anti-CMV IgM. It is difficult to ascertain whether these infants were infected postnatally although the anti-CMV IgG levels at 1-month old were lower than in the cord blood. Nevertheless, according to the seroconversion of CMV IgM and IgG, and the dynamic changes of IgG titers (decreasing, followed by increasing) in infants [23], we can get the definitive diagnosis of primary CMV infection at least in most of the infants in the present study.

In view of the most CMV infection in children occurred before 3.5-month old, Chinese children may acquire CMV too early to be prevented by vaccination. Therefore, we consider that children, at least in developing countries, should not be the target population for the future licensed vaccine against CMV infection.

## Methods

### Subjects and serum specimens

The subjects recruited in this investigation included two groups of children, and each child had a different family. A retrospective group was composed of 112 singleton full-term newborns and their 112 mothers. All mothers’ sera and cord blood samples, and longitudinal samples from 41 infants at ages of 1, 3.5, 8, and 24 months respectively were prospectively collected in a study on hepatitis B vaccine conducted in Nanjing Drum Tower Hospital from December 2006 to April 2009 [24]. The mothers were 20–36 years of age (mean, 27.8 ± 2.6), 68 with vaginal delivery and 44 with caesarean section. The male newborns were 59. All newborns were in good health based on their birth records, with Apgar scores >8 and body weights 2800–4600 g (3450.7 ± 369.6), and had no symptoms related with CMV infection after birth and during the follow-up. All the children did not attend daycare since it is a widely accepted custom in China that grandparents help to take care of their grandchildren.

The other group was composed of children who underwent conventional physical examination in Nanjing Children’s Hospital, June–September 2011. They were recruited to investigate anti-CMV IgG seroprevalence among children. We explained to the mothers/fathers or guardians that their children’s remained sera after laboratory tests would be used for this study. After getting the written informed consent from the mothers/fathers or guardians and excluding those with abnormal symptoms or laboratory tests, such as fever, congenital malformations and liver function abnormalities, a total of 837 sera from 837 children (aged 0–8 years, 513 boys), who were in good health and had no symptoms related with CMV infection, were collected.

This study was approved by the institutional review boards of Nanjing Drum Tower Hospital (2011OG02NDTH) and Nanjing Children’s Hospital (NCH2011518). For the retrospective cohort, since the mothers consented in the other study [24], mothers’ and children’s serum samples were used in the present study via an exemption approved by the institutional review board of Nanjing Drum Tower Hospital. For the cross-sectional group, mothers/fathers or guardians gave the written informed consent for the use of their children’s sera.

### Quantification of anti-CMV IgG

Sera were quantitatively tested for anti-CMV IgG using a commercial enzyme-linked immunosorbent assay (ELISA) kit (Dia.Pro Diagnostic Bioprobes, Milano, Italy) [25-27]. The kit contains the microplate coated with native CMV antigens, which were inactivated and highly purified by sucrose gradient centrifugation. Additionally, the kit contains human plasma derived calibrators with anti-CMV IgG at concentrations of 0, 0.5, 1, 2, 4, and 8 IU/ml, which were titrated on WHO standard (proposed international standard). In the measurement, paired maternal and cord sera, firstly diluted 1:101 with diluent (2% casein, 10 mM Tris-citrate buffer and 0.1% Tween 20), were tested in parallel, and positive and negative controls provided in the kit were also included. Calibration curves were established for each test. Based on the manufacturer’s instruction, the diluted sample with a concentration >0.5 IU/ml was considered positive for anti-CMV IgG, while the sample with a concentration ≤0.5 IU/ml was considered negative. When the IgG concentration was beyond the upper detection limit (8 IU/ml), the sera were retested by further dilution. Each test showed good linearity.

### Detection of anti-CMV IgM

Anti-CMV IgM was measured by the CMV IgM capture immunoassay (Dia.Pro Diagnostic Bioprobes). The kit contains the microplate coated with anti-human IgM, which was derived from mouse monoclonal antibody and purified by affinity chromatography. Anti-CMV IgM positive and negative human plasma controls were provided in the kit. In each measurement, serum samples were diluted same as in detection of anti-CMV IgG, and positive and negative controls were included. As recommended by the manufacturer, the cut-off value was calculated as follows: cut-off = OD450 for negative control + 0.250. The test result was interpreted as a ratio of the sample OD450 and the cut-off value (S/Co). The sample was considered positive if S/Co value was >1.2, indeterminate if it was 1.0–1.2, and negative if it was <1.0. The indeterminate sample was retested; the sample was considered positive if S/Co value was 1.0–1.2 or >1.2, and negative if it was <1.0.

### CMV IgG avidity assay

CMV IgG avidity index (AI) was measured by 8 M urea denaturation procedures using CMV IgG ELISA kit (Dia.Pro Diagnostic Bioprobes) as previously reported [28,29]. Briefly, for each sample, a 50 μl aliquot of appropriately diluted serum was added to CMV antigen-coated wells in duplicate [30]. Then 50 μl 8 M urea and 50 μl specimen diluent (2% casein, 10 mM Tris-citrate buffer pH 6.0, 0.1% Tween 20) were applied to the denaturation and reference wells respectively. The assay was then continued according to the manufacture’s recommendations. For a given serum, the AI was calculated as follows: (net OD in the presence of urea/net OD in the absence of urea) × 100%. An AI lower than 30% was considered as low, between 30% and 50% as intermediate, and greater than 50% as high-avidity antibodies.

### Statistical analysis

Data were analyzed with SPSS version 17.0. Statistical comparisons of the IgG levels between different groups were analyzed by *t*-test. Chi-squared test or Fisher’s exact test was used for categorical data. Linear regression analysis was performed in correlating maternal and cord blood titers. A *p* value less than 0.05 was considered statistically significant.

## Abbreviations

CMV: Cytomegalovirus; IgG: Immunoglobulin G; IgM: Immunoglobulin M; ELISA: Enzyme-linked immunosorbent assay; AI: Avidity index; GMC: Geometric mean concentration.

## Competing interests

The authors declare that they have no competing interests.

## Authors’ contributions

YH, YHZ and JC were responsible for the conception and design of the study. MW and TZ were involved in blooding sampling and collecting relevant information. Acquisition of data and interpretation of data were carried out by YHZ, JC and LH. JC undertook the statistical analysis and wrote the first draft of the paper, in collaboration with LH, MW and TZ. All authors contributed to revise the article critically for important intellectual content and have approved the final version of the manuscript to be published. YH and YHZ act as the guarantor of the study.
